# The effect of propolis oral care on oral mucositis in intensive care patients with chronic obstructive pulmonary disease: a randomized controlled trial

**DOI:** 10.1590/1806-9282.20260772

**Published:** 2026-10-30

**Authors:** Büşra Nur Özel Akyürek, Sevda Korkut, Mustafa Öner Küçük, Ayşe Işın Gürpınar

**Affiliations:** 1 Erciyes University, Institute of Health Sciences, Department of Nursing – Kayseri, Türkiye.; 2 Erciyes University, Faculty of Health Sciences, Department of Nursing – Kayseri, Türkiye.; 3 Nuh Naci Yazgan University, Faculty of Dentistry, Department of Microbiology – Kayseri, Türkiye.; 4 Kayseri State Hospital, Department of Chest Diseases – Kayseri, Türkiye.

**Keywords:** COPD, Nursing, Oral mucositis, Propolis

## Abstract

**OBJECTIVE:**

This study aimed to evaluate the preventive effect of adding a propolis gargle to standard oral care on the development of oral mucositis in critically ill patients with chronic obstructive pulmonary disease.

**METHODS:**

This randomized controlled trial included 60 critically ill patients with chronic obstructive pulmonary disease. Both groups received the institutional standard oral care protocol with 0.2% chlorhexidine gluconate three times daily. The intervention group additionally received a propolis gargle three times daily for 15 days. Oral mucosal status was evaluated using the total oral mucosa score.

**RESULTS:**

There was no significant difference in oral mucosa scores between the groups at baseline, whereas oral mucosa scores were significantly lower in the intervention group than in the control group at the 7- and 15-day follow-ups (p<0.001).

**CONCLUSION:**

Adding propolis gargle to standard oral care significantly reduced the development of oral mucositis in critically ill patients with chronic obstructive pulmonary disease. However, because the intervention group received additional oral care exposure, the independent effect of propolis cannot be determined and requires further investigation.

**Clinical Trial Registration Number**: ClinicalTrials.gov Identifier: NCT07426198.

## INTRODUCTION

Chronic obstructive pulmonary disease (COPD), a significant public health problem worldwide in terms of both morbidity and mortality, ranks third among the leading causes of death today^1^. Characterized by progressive airway obstruction, COPD causes persistent respiratory symptoms, and the clinical symptoms progressively worsen as the disease advances^2^. Increased dyspnea limits individuals’ daily activities and causes significant losses in psychological and social functioning, along with a decrease in physical capacity^1,3^. The chronic progression of the disease, complex treatment processes, and side effects associated with long-term medication use can make it difficult for individuals to meet their self-care needs and may lead to inadequate oral care. This condition may be associated with the prevalence of oral mucosal problems in individuals with COPD^3^. These challenges may become particularly relevant in the ICU, where critically ill patients frequently have substantial oral care needs and may depend on nursing care to maintain adequate oral hygiene^4^.

Numerous risk factors negatively affect oral mucosal integrity in individuals with COPD. Long-term inhaler and systemic medication use, as well as oxygen therapy, can lead to decreased saliva secretion and structural changes in the oral mucosa^5^. In addition, advanced age, smoking, nutritional deficiencies, and inadequate oral hygiene habits disrupt the oral microbiota balance and cause damage to the oral mucosal integrity^6^. One of the clinically important consequences of impaired oral mucosal integrity is the development of oral mucositis^7^. Oral mucositis can adversely affect essential functions such as eating, swallowing, speaking, and maintaining adequate fluid intake, thereby negatively impacting quality of life^8,9^.

Various pharmacological and supportive approaches have been described in the literature to preserve oral mucosal integrity, prevent the development of mucositis, or treat established mucositis^10-13^. Topical applications of vitamins A and E, glutamine, honey, aloe vera, omega-3 fatty acids, black mulberry, and propolis are also reported among supportive approaches for preventing oral mucositis and alleviating its symptoms^14,15^. However, the available evidence is heterogeneous, and much of the clinical evidence concerning propolis and oral mucositis has been generated in oncology populations. Studies conducted particularly in oncology populations have reported favorable outcomes associated with propolis-containing interventions, including reductions in the development or severity of oral mucositis^10^.

Due to its high phenolic content, propolis exhibits anti-inflammatory, antioxidant, antibacterial, antiviral, and antifungal properties^16,17^. The biological rationale for investigating propolis may also be considered within the conceptual framework of the Sonis model of mucositis pathobiology, which describes mucosal injury as a dynamic process involving initiation, inflammatory signaling and amplification, ulceration, and healing. Although this model was developed primarily in the context of cancer therapy-induced mucositis, it provides a conceptual basis for considering how the anti-inflammatory and antioxidant properties of supportive agents such as propolis may be relevant to mucosal protection^18^. Nevertheless, evidence regarding propolis-containing oral care in patients with COPD receiving intensive care is limited. Given the vulnerability of these patients to deterioration in oral mucosal integrity, evaluating supportive oral care strategies in this population is clinically relevant. Therefore, this study aimed to evaluate the preventive effect of adding a propolis gargle to standard oral care on the development of oral mucositis in critically ill patients with COPD.

### Research hypotheses

H_1_: Adding a propolis gargle to standard oral care in critically ill patients with COPD reduces the frequency and severity of oral mucositis compared to standard oral care alone.

## METHODS

### Study design

This study was conducted as a randomized controlled trial. The CONSORT checklist was used to report the study.

### Study population and sample

The study population consisted of patients receiving COPD treatment in the intensive care unit of a tertiary care hospital between January 1, 2025, and June 10, 2025. The sample size was calculated using G*Power version 3.1.9.7 based on oral assessment scores reported in a previous study^19^. With a Type I error rate of 5% (α=0.05), a statistical power of 95% (1-β=0.95), and an effect size of 0.92, the minimum required sample size was 27 participants per group. To account for potential participant attrition, the target sample size was set at a minimum of 30 participants per group. The study was completed with 60 patients (Figure 1).

**Figure 1:**
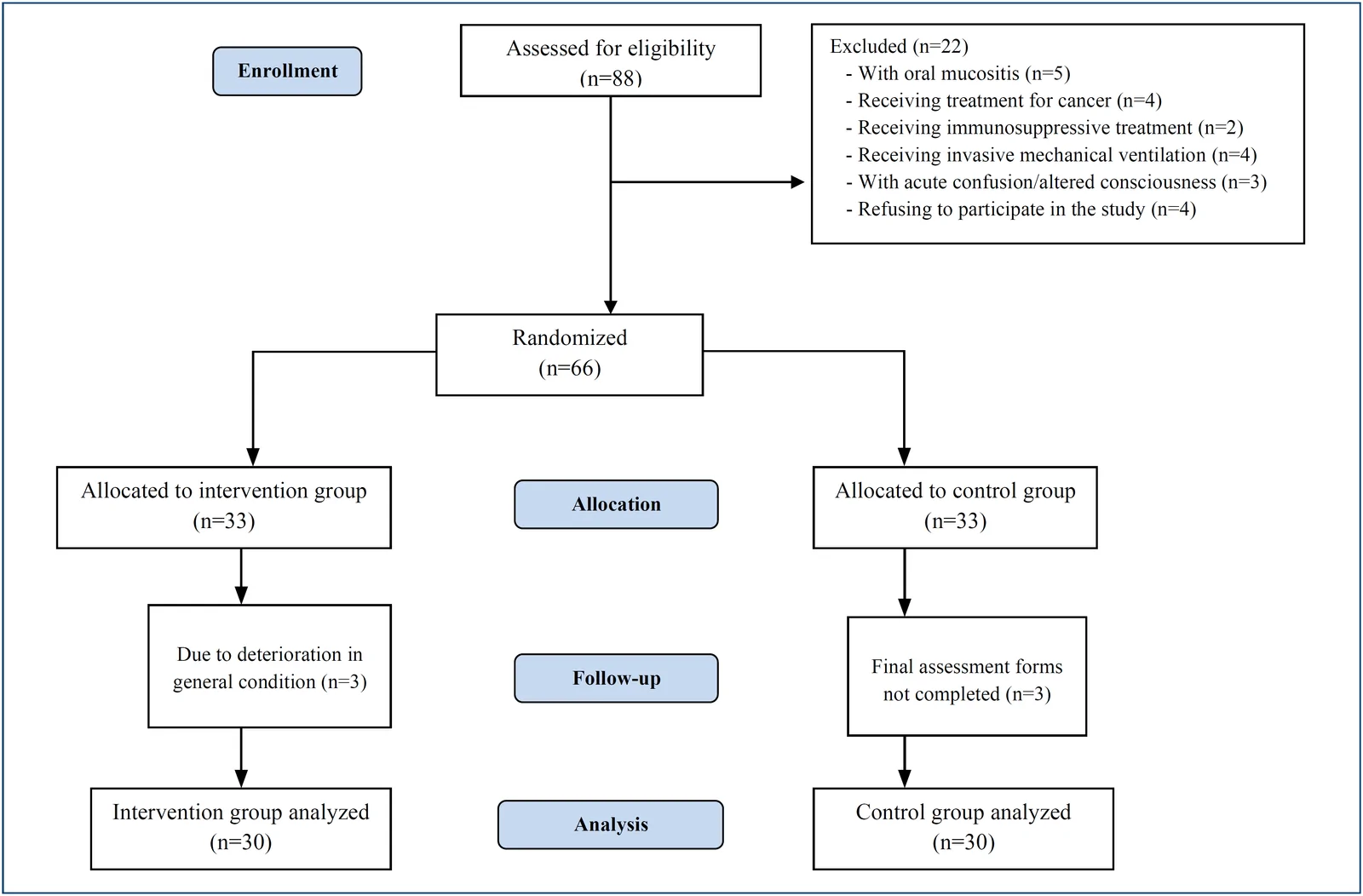
CONSORT flow diagram.

The inclusion criteria were as follows: (1) aged 18 years or older; (2) diagnosed with COPD; (3) no oral mucositis at baseline; (4) no known allergy to bees or bee products; (5) able to speak and understand Turkish and communicate adequately with the researcher; (6) conscious and able to understand and safely perform the gargling procedure, including retaining the solution in the oral cavity and expectorating it after application; (7) without a psychiatric illness or a mental or physical condition that would prevent participation in the study procedures; and (8) willing to participate. The exclusion criteria were as follows: (1) cancer diagnosis, (2) immunosuppressive therapy, (3) invasive mechanical ventilation, and (4) altered consciousness or acute confusion that prevented safe performance of the gargling procedure. Non-invasive ventilation (e.g., BiPAP) was not an exclusion criterion provided that the patient was conscious, cooperative, and able to perform the gargling procedure safely.

### Data collection tools

Information form: This form, developed by the researchers, includes questions regarding participants’ sociodemographic characteristics and disease-related information.

Oral assessment guide: This assessment guide evaluates oral mucosal integrity through visual examination and subjective information reported by the individual. It addresses changes in the oral mucosa in eight different dimensions. These dimensions include changes observed in the voice, swallowing, lips, tongue, saliva, mucous membrane, gums, and teeth or dentures. Each assessment area is scored on a scale of 1, 2, or 3 points based on the severity of changes in the oral mucosa. All points obtained from the eight areas are added together to calculate the total oral mucosa score (OMS). Higher total scores reflect greater severity of oral mucosal impairment and associated clinical manifestations^20^.

### Data collection

Data were collected through face-to-face interviews with participants in the intervention and control groups. Participants who agreed to participate in the study were provided with verbal and written information regarding the study's purpose, scope, and procedures. Written informed consent was obtained from all participants before enrollment.

Following completion of the baseline assessments, randomization and group allocation were performed by a statistician using a computer-generated randomization list, with stratification based on gender and COPD stage. The researcher was not informed of the participant's group assignment until the allocation had been completed. After allocation, the researcher became aware of group assignment because of involvement in the implementation and monitoring of the intervention protocol.

At baseline, the Descriptive Information Form and Oral Assessment Guide were administered. Data collection forms were completed by the researcher based on medical record review, information obtained from the participants, and oral examination.

Participants in both groups received the institutional standard oral care protocol with 0.2% chlorhexidine gluconate (CHG) three times daily at eight-hour intervals. In addition to standard CHG oral care, participants in the intervention group received a propolis gargle three times daily for 15 days. A commercially available, water-soluble propolis preparation containing 10% pure Anatolian propolis was used in the intervention. For each application, 10 drops of propolis were added to a cup of water, and participants gargled with the solution for one minute. The propolis gargle was administered one hour after routine CHG oral care. Thus, the intervention group received three additional oral rinsing procedures per day compared with the control group.

The oral care applications were performed by the researcher or clinical nurses who had been trained in the study protocol. Before initiation of the study, all nurses working in the participating intensive care units received verbal and written training regarding the purpose, dose, frequency, and administration of propolis oral care. Intervention delivery was monitored using a standardized application form, on which the timing and duration of each application and any potential adverse effects were recorded. The researcher regularly monitored adherence to the intervention protocol throughout the follow-up period. All scheduled propolis applications were completed, and no adverse reactions requiring discontinuation of the intervention were observed.

Participants in both groups were assessed on days 1, 7, and 15 using the Oral Assessment Guide. The researcher who performed the oral mucosal assessments was not blinded to group allocation, as the researcher was also involved in the implementation and monitoring of the intervention protocol. Oral mucosal assessments were performed by the same researcher using standardized assessment tools and procedures at each follow-up time point.

### Data analysis

Statistical analyses were conducted using IBM SPSS Statistics for Windows, version 25.0 (IBM Corp., Armonk, NY, USA). The normality of continuous variables was assessed using histograms, Q–Q plots, and the Shapiro-Wilk test. The chi-square test was used to compare the descriptive characteristics. The Mann-Whitney U test was applied to compare OMS scores. The Friedman test was used to evaluate changes across intra-group follow-up times, and when significant differences were detected, post-hoc pairwise comparisons were performed using the Bonferroni correction to control for the risk of Type I error. A significance level of p<0.05 was accepted.

### Ethical considerations

Ethical approval was obtained from the GETAT Clinical Research Ethics Committee (Ethics Committee Decision No: 73, Date: 09.05.2024). In addition, written institutional permission was obtained from the hospital. Approval was obtained from the Ministry of Health's General Directorate of Health Services to conduct the research. Individuals who agreed to participate in the study were provided with detailed information about the study and were asked to sign an Informed Consent Form. Because propolis was not part of the institutional standard oral care protocol and may cause hypersensitivity reactions in susceptible individuals, patients with a known allergy to bees or bee products were excluded from the study. Participants receiving propolis were monitored throughout the intervention for potential allergic reactions, oral irritation, burning, or sensitivity, and discontinuation of the intervention was planned in the event of an adverse reaction.

## RESULTS

No statistically significant differences were observed between the intervention and control groups with respect to gender, age group, educational level, income status, body mass index, smoking status, COPD stage, comorbidities, or medication use (p>0.05). These results indicate that the research groups were similar in terms of their initial characteristics (Table 1).

**Table 1:** Introductory characteristics of individuals in the intervention and control groups.

Characteristics		Intervention group (n=30)		Control group (n=30)		Test p-value*
		n	%	n	%	
Gender						
Female		9	30.0	10	33.3	χ^2^=0.000 p=1.000
Male		21	70.0	20	66.7	
Age groups						
	50–59 years	4	13.3	5	16.7	χ^2^=0.666 p=0.770
	60–69 years	8	26.7	10	33.3	
	70 years and over	18	54.5	15	50.0	
Educational status						
	Illiterate	7	23.3	5	16.7	χ^2^=0.420 p=0.811
	Primary school	13	43.3	14	46.7	
	Secondary school/high school	10	33.4	11	36.7	
Income status						
	Low	22	73.3	18	60.0	χ^2^=0.675 p=0.412
	Middle	8	26.7	12	40.0	
Body mass index (BMI)						
	Normal (20–24.9)	5	16.7	6	20.0	χ^2^=0.615 p=0.778
	Overweight (25–29.9)	18	60.0	15	50.0	
	Obese (30 and above)	7	23.3	9	30.0	
Smoking status						
	Current smoker	16	53.3	16	53.3	χ^2^=0.221 p=1.000
	Never smoker	5	16.7	6	20.0	
	Former smoker	9	30.0	8	26.7	
COPD stage						
	Stage 1	2	6.7	3	10.0	χ^2^=0.440 p=1.000
	Stage 2	9	30.0	8	26.7	
	Stage 3	16	53.3	16	53.3	
	Stage 4	3	10.0	3	10.0	
Additional chronic disease status						
	Yes	26	86.7	26	86.7	χ^2^=0.000 P=1.000
	No	4	13.3	4	13.3	
Medications used**						
	Oxygen therapy	14	46.7	13	43.3	χ^2^=25.340 p=0.646
	Bronchodilator	25	83.3	19	63.3	
	Corticosteroid	9	30.0	6	20.0	
	Antihypertensive	8	26.7	8	26.7	
	Antidiabetic	7	23.3	7	23.3	
	Other***	11	36.7	9	30.0	

* Chi-square analysis.

** Multiple answers were given.

*** Thyroid medications, rhythm regulators, lipid regulators, etc. BMI: body mass index; COPD: chronic obstructive pulmonary disease.

While there was no statistically significant difference between the groups’ OMSs on day 1 (p>0.05), a significant difference was observed between the intervention and control groups on days 7 and 15 (p<0.001). In the within-group analyses, a significant decrease in OMSs was observed in the intervention group on day 15 (p<0.001), while in the control group, OMSs increased significantly on day 7 compared to day 1, and no significant difference was found between day 7 and day 15 (p<0.001) (Table 2).

**Table 2:** Distribution of oral mucosa scores for individuals in the intervention and control groups according to intergroup and intragroup follow-up times.

Groups	Day 1	Day 7	Day 15	Test p-value
	Median (25–75%)	Median (25–75%)	Median (25–75%)	
Intervention group (n=30)	11.5 (11.0–12.2)^a^	11.0 (10.7–12.2)^a^	9.0 (9.0–10.0)^b^	42.389** <0.001
Control group (n=30)	11.5 (11.0–13.0)^a^	14.0 (13.0–15.2)^b^	15.0 (14.0–16.0)^b^	35.261** <0.001
Test p-value	434.500* 0.814	97.500* <0.001	12.500* <0.001	

* Mann-Whitney U test.

** Friedman analysis.

a,b The same letters indicate no difference between groups, while different letters indicate a difference.

All scheduled propolis applications were completed during the intervention period, and no adverse events related to the propolis gargle, including allergic reactions, oral irritation, burning, or sensitivity, were observed.

## DISCUSSION

The study demonstrated that the addition of a propolis gargle to the standard oral care protocol significantly reduced the frequency and severity of oral mucositis compared with standard oral care alone. The lower OMSs observed over time in the intervention group suggest that the combined oral care protocol may contribute to maintaining oral mucosal integrity and limiting mucosal deterioration. In contrast, the increase in OMSs in the control group indicates that standard oral care alone may not have been sufficient to prevent progressive oral mucosal deterioration in this sample of critically ill patients with COPD. This finding is consistent with evidence from intensive care settings emphasizing the importance of systematic oral assessment and structured oral care in maintaining oral health in critically ill patients^4,21^. Previous ICU studies have also indicated that the effectiveness of oral care may depend not only on the oral care agent used but also on how frequently and systematically care is delivered^22,23^. Thus, the present findings should be considered within the broader context of oral care delivery rather than attributed solely to the specific product used.

Propolis has anti-inflammatory and antioxidant properties that provide a biologically plausible explanation for a potential contribution to preservation of oral mucosal integrity^24^. Consistent with this possibility, Çakmak and Nural reported beneficial effects of propolis on oral mucositis in patients undergoing high-dose chemotherapy^10^. However, these findings cannot be directly extrapolated to the present ICU population because the clinical context, underlying disease, and oral mucositis risk factors differ. More importantly, the present design does not permit the observed between-group difference to be attributed specifically to the pharmacological properties of propolis. Participants in the intervention group received the routine three-times-daily CHG oral care plus three additional propolis gargles per day, whereas participants in the control group received only routine CHG oral care. Therefore, the intervention differed from the control condition in both oral care agent and additional oral care exposure. The favorable outcomes may consequently reflect the pharmacological properties of propolis, the effect of additional oral rinsing and oral care exposure, or a combination of these factors.

This methodological issue is particularly relevant when interpreting the findings in relation to other oral care interventions in intensive care. Evidence from ICU settings indicates that oral care should be individualized according to patients’ oral care needs and that the frequency of oral care itself may influence oral mucosal outcomes^22,23^. Accordingly, this study provides evidence for the combined intervention strategy—standard CHG oral care supplemented with a propolis gargle—rather than for an isolated effect of propolis. This two-group design limits our ability to determine the extent to which the observed differences can be specifically attributed to propolis. Future studies should employ more rigorous randomized controlled designs to further clarify the specific contribution of propolis to the observed effects and to account for potential confounding factors related to the oral care protocol.

### Limitations

This study has several limitations. The single-center design and limited sample size may restrict the generalizability of the findings. The 15-day follow-up period precluded evaluation of longer-term outcomes, and the inclusion of only patients with COPD limits generalization to other patient populations. Importantly, the intervention group received three additional propolis gargles per day in addition to routine CHG oral care, whereas the control group received only routine CHG oral care. Therefore, the independent effect of propolis cannot be distinguished from the potential effect of increased oral care frequency. Future studies using frequency-matched control conditions, including an appropriate placebo gargle, are needed to isolate the specific contribution of propolis.

In addition, because the propolis intervention required participants to actively gargle and expectorate the solution, only conscious and cooperative patients capable of safely performing this procedure were included. Consequently, the findings may not be generalizable to critically ill patients with COPD who have impaired consciousness, acute confusion, or are otherwise unable to perform active oral rinsing procedures.

The lack of assessor blinding is another limitation of this study. Because the researcher performing the oral mucosal assessments was aware of group allocation, observer bias cannot be excluded, particularly because the Oral Assessment Guide includes visual and subjective components. Future studies should incorporate blinded outcome assessment whenever feasible.

## CONCLUSION

Adding propolis gargle to standard oral care significantly reduced the development of oral mucositis in critically ill patients with COPD. However, because the intervention group received additional oral care exposure, the independent effect of propolis cannot be determined. Further randomized studies using frequency-matched control conditions are needed to clarify the specific prophylactic effect of propolis.

## Data Availability

The datasets generated and/or analyzed during the current study are available from the corresponding author upon reasonable request.
